# Transcriptomic Reprogramming, Alternative Splicing and RNA Methylation in Potato (*Solanum tuberosum* L.) Plants in Response to Potato Virus Y Infection

**DOI:** 10.3390/plants11050635

**Published:** 2022-02-25

**Authors:** Anna Glushkevich, Nadezhda Spechenkova, Igor Fesenko, Andrey Knyazev, Viktoriya Samarskaya, Natalia O. Kalinina, Michael Taliansky, Andrew J. Love

**Affiliations:** 1 Shemyakin-Ovchinnikov Institute of Bioorganic Chemistry of the Russian Academy of Sciences, 117997 Moscow, Russia; zverochek2@gmail.com (A.G.); rysalka47@gmail.com (N.S.); fesigor@gmail.com (I.F.); agrofak@gmail.com (A.K.); viktoriya.samarskaya2012@yandex.ru (V.S.); 2 Belozersky Institute of Physico-Chemical Biology, Lomonosov Moscow State University, 119991 Moscow, Russia; kalinina@belozersky.msu.ru; 3 The James Hutton Institute, Invergowrie, Dundee DD2 5DA, UK

**Keywords:** potato virus Y, heat stress, complex stress, direct RNA-seq, lncRNAs, PARylation

## Abstract

Plant-virus interactions are greatly influenced by environmental factors such as temperatures. In virus-infected plants, enhanced temperature is frequently associated with more severe symptoms and higher virus content. However, the mechanisms involved in controlling the temperature regulation of plant-virus interactions are poorly characterised. To elucidate these further, we analysed the responses of potato plants cv Chicago to infection by potato virus Y (PVY) at normal (22 °C) and elevated temperature (28 °C), the latter of which is known to significantly increase plant susceptibility to PVY. Using RNAseq analysis, we showed that single and combined PVY and heat-stress treatments caused dramatic changes in gene expression, affecting the transcription of both protein-coding and non-coding RNAs. Among the newly identified genes responsive to PVY infection, we found genes encoding enzymes involved in the catalysis of polyamine formation and poly ADP-ribosylation. We also identified a range of novel non-coding RNAs which were differentially produced in response to single or combined PVY and heat stress, that consisted of antisense RNAs and RNAs with miRNA binding sites. Finally, to gain more insights into the potential role of alternative splicing and epitranscriptomic RNA methylation during combined stress conditions, direct RNA nanopore sequencing was performed. Our findings offer insights for future studies of functional links between virus infections and transcriptome reprogramming, RNA methylation and alternative splicing.

## 1. Introduction

Potato (*Solanum tuberosum* L.), one of the most important non-grain crops in the world, similar to all other crop plants, is constantly exposed to a plethora of pathogens. Among these pathogens, viruses account for up to 50% of all novel and emerging plant diseases [1]. Potato virus Y (PVY) is one of the most important pathogens of potato, which has a significant negative impact on potato-crop yield and quality [2,3]. To help prevent such crop losses, it is essential that we improve plant resistance mechanisms against viruses, which constitutes the most efficient and reliable strategy for plant protection.

Plant resistance to viruses is multifaceted and involves many different mechanisms which are governed by the type of virus and host-plant species. Briefly, a first layer of innate immunity against viruses (as well as against other pathogens) is the recognition of pathogen-associated molecular patterns (PAMPs) by pattern recognition receptors (PRRs) at the plasma membrane, which leads to the induction of defence signalling and causes PAMP-triggered immunity (PTI) [4,5]. It has recently been suggested that dsRNAs associated with virus replication may also act as conserved molecular patterns which represent genuine PAMPs in infected plants and that these may associate with cytoplasmic or membrane-bound PRRs to trigger PTI [4,5]. PTI defence induction results in the accumulation of various signalling components, such as Ca^2+^, reactive oxygen species (ROS), mitogen-activated protein kinase (MAPK) cascades, hormones (including salicylic acid (SA), jasmonic acid (JA), ethylene (Et) and abscisic acid (ABA)) and the enhanced expression of a number of defence genes such as those encoding nucleotide-binding and leucine-rich repeat (NB-LRR disease resistance) proteins and pathogenesis-related proteins, which can culminate in reduced or blocked pathogen invasion [4,6,7,8]. A second layer of immune response occurs in plants carrying resistance (*R* or *N*) genes that employ effector-triggered immunity (ETI). This typically involves the interaction between virus-derived effectors and host resistance R or N (mostly NB-LRR) proteins that trigger a number of intracellular signalling events, which leads to disease resistance [9]. For example, in potato varieties carrying a *Ny* resistance gene, there is a PVY strain-specific hypersensitive response (HR) or programmed cell-death activation which leads to the rapid necrotization of tissue around the virus and prevents further pathogen spread. Different potato varieties may also possess various *Ry* resistance genes which can provide extreme resistance (ER) to a broad range of PVY strains and typically results in very rapid restriction of the virus to only a few epidermal cells [10,11,12]. The complex molecular mechanisms of HR and ER against PVY are described in detail in [9]. When host defences successfully prevent virus invasion, these types of interaction are referred to as incompatible plant-virus interactions.

In contrast to incompatible responses, compatible infections of plants result in successful systemic invasion, particularly when the host plant contains no *R* or *N* resistance genes against the virus. In such scenarios, although the virus replicates and spreads *in planta*, the plant may still sense the presence of the pathogen and may invoke defence responses associated with aspects of incompatibility such as PTI-based responses (which can be triggered via virus-specific dsRNA; [4,5]), although these do not completely halt invasion. Another important factor conferring antiviral resistance in compatible plant-virus interactions is based on RNA interference (RNAi) or RNA silencing. RNAi is a ubiquitous nucleotide sequence-specific gene-regulation mechanism involving the generation of small RNAs that target the silencing machinery to complementary DNA or RNA for transcriptional (TGS; methylation) or post-transcriptional (PTGS; degradation or repression of virus genome translation) silencing, respectively [13,14,15,16]. In the context of RNA viruses, dsRNA molecules formed during virus replication are recognised and cleaved by Dicer-like proteins (DCL) into small interfering RNAs (siRNAs). siRNAs are loaded into the RNA-induced silencing complex containing members of the ARGONAUTE (AGO) protein family, which directs it to specifically degrade complementary viral RNAs [13,14,15,17,18,19]. This can result in the prevention of viral invasion of previously uninvaded tissues during an initially compatible infection.

Interestingly, plant antiviral-resistance mechanisms may also be regulated via the functional interplay between viruses and the methionine cycle (MTC; [20,21,22]). We previously demonstrated that PVY infection generally upregulates the accumulation of major MTC enzymes in PVY-resistant potato cv Gala, leading to a significantly increased accumulation of S-adenosyl methionine (SAM), a key component of MTC which acts as a universal methyl donor in trans-methylation reactions. In contrast to this, in PVY-susceptible cv. Chicago, SAM levels were not increased by PVY, which correlated with the enhanced susceptibility to PVY. These data suggest that MTC and its major transmethylation function may determine potato resistance or susceptibility to PVY.

There are also several other important host-encoded factors which affect resistance to plant viruses and PVY in particular. Among them are coilin, a structural protein of subnuclear Cajal bodies which mediates the defence response against tobacco rattle virus (TRV; [23,24]) but facilitates susceptibility to PVY [23] and major latex protein (MLP), which induces protection against PVY [25].

In nature, plants, including potato crops, not only face an onslaught of biotic stress invoked by pathogen exposure, but they are often simultaneously exposed to various environmental stresses, such as elevated temperatures. Increased temperatures are particularly detrimental to potatoes, which is a cool-weather crop and has optimal growth at temperatures between 14 and 22 °C; above these temperatures, its yield is significantly reduced, which is likely to become an increasing problem given climate change expectations [26].

To cope with higher temperatures, plants have evolved mitigation mechanisms for the acquisition of thermotolerance. Heat stress may invoke the reprogramming of various signalling pathways, such as the production of reactive oxygen species (ROS), induction of mitogen-activated calcium-dependent protein kinase signal transduction cascades, increased expression of a variety of heat shock transcription factors (HSFs), heat shock proteins (HSPs) and accumulation of osmolytes that affect membrane fluidity and morphology [27]. HSPs constitute a family of proteins including HSP90, HSP70 and small HSPs (such as HSP20), which are responsible for protein folding, assembly, stabilization, translocation and degradation in many normal cellular processes. It has been suggested that the regulation and maintenance of thermotolerance in plants is a complex multifactorial process and a variety of plant hormones such as salicylic acid (SA), have been implicated in these processes [27].

It is well accepted that environmental cues including heat can significantly affect interactions of viruses with their plant hosts [28]. A broad range of defence responses which can break down at high temperatures have been reported, particularly for incompatible interactions such as *R* or *N* gene-mediated resistance. Most of these potato resistance genes, such as *Ny-1* in potato cultivar Rywal and *Ny* found in *S. sucrense* and *S. sparsipilum*, confer resistance only at low temperatures (16–20 °C). At higher temperatures (24–28 °C) resistance does not develop, and PVY spreads systemically throughout the plant [29,30].

Compatible plant–virus interactions may also be influenced by heat stress. For example, tomato plants and Arabidopsis exposed to heat stress were, respectively, more sensitive to tomato yellow leaf curl virus (TYLCV) [31] and turnip mosaic virus (TuMV) [28], respectively. Similarly, high temperatures significantly increased the susceptibility of potato cv Chicago to PVY [21,32]. In the latter case, it was shown that heat stress induced changes in the level of MTC metabolites, which suggested that the enhanced susceptibility of potato plants to PVY under these conditions may be partly orchestrated by the downregulation of MTC enzymes and resultant cycle perturbations. In line with this, the topical treatment of these plants with methionine restored the accumulation of MTC metabolites and reversed the susceptibility to PVY at elevated temperatures.

In contrast, the RNA silencing-mediated defence is facilitated by rising temperatures, which may concomitantly reduce the development of virus diseases [33,34,35]. This situation is highly complex given that RNA silencing and virus-encoded silencing suppressors are associated with phytohormone-mediated defence pathways, which can also in turn be regulated by temperatures [36,37,38,39,40]. Together, this evidence suggests that particular components of the complex plant defence system may govern different virus-plant combinations and that they may be further differentially regulated by elevated temperatures.

The molecular pathways induced in responses to virus infections and heat stress can overlap each other and the mechanisms controlling the resistance or susceptibility of plants to a virus under elevated temperatures may be activated in response to this combined stress as a result of the integration of individual stress-responsive signalling cascades [41].

To shed more light on interactive molecular responses to combined heat stress and virus infection in potato, a comparative transcriptomic (RNAseq) analysis of single and combined stress responses was carried out in potato cv Chicago. In addition to protein-coding RNA transcripts, the expression of long non-coding RNAs (lncRNAs), which have been shown to play various key roles in different biological processes, [42] was analysed. Finally, to gain more insights into the potential role of alternative splicing and epitranscriptomic RNA methylation during combined stress conditions, direct RNA nanopore sequencing was performed.

## 2. Results and Discussion

### 2.1. Impact of Elevated Temperature on the Susceptibility of Potato Plants (cv Chicago) to PVY

In previous work we studied the effect of a moderately elevated temperature (28 °C) on the dynamics of PVY infection in potato plants [32], a temperature which mimics the mild heat-stress conditions that may arise as a result of global climate change. In inoculated leaves of cv Chicago, PVY was detected at 3 days post inoculation (dpi) at low levels, which did not significantly increase with time or temperature variation [32]. Starting at approximately 8 dpi, PVY spread systemically, invading upper leaves at both normal (22 °C) and elevated (28 °C) temperatures. With time, an increase in virus titer was observed in the systemically infected leaves of plants grown at 22 °C (up to seven-fold); however, virus levels were found to be significantly higher in corresponding tissues of plants grown at 28 °C [32], suggesting that rising temperature greatly enhances the susceptibility of Chicago plants to PVY.

More recently, we used isobaric tag for relative and absolute quantitation (iTRAQ) labeling to comprehensively analyze changes in the proteome of potato plants (cv Chicago) infected with PVY at normal (22 °C) and elevated temperature (28 °C) conditions [21]. At an elevated temperature, the proteome changes were much more pronounced. Proteins in the upregulated group were mainly associated with general host responses to plant stress and disease, but the most striking finding was the downregulation of enzymes attributed to MTC which led us to the suggestion that MTC and trans-methylation play the important role in plant-PVY interactions.

In the present work, we expand on the previous study to further examine transcriptomic changes in PVY-infected Chicago plants. In all PVY-infected plants used in this work, which were maintained at both normal (22 °C) and elevated (28 °C) temperatures, the virus invaded non-inoculated (systemically infected) leaves as early as 8 dpi, with essentially similar PVY RNA loads irrespective of temperature (see Section 2.5). However, a significant rise in PVY levels occurred at 28 °C (compared with 22 °C) at the later stages of infection. This suggests that 8 dpi may represent a critical time point in determining the temperature sensitivity of potato plants to PVY, since PVY accumulation undergoes a temperature-dependent divergence beyond this time point. Consequently, this time point was selected for further RNAsec analysis.

### 2.2. Transcriptome Analysis: Overview

**Figure 1:**
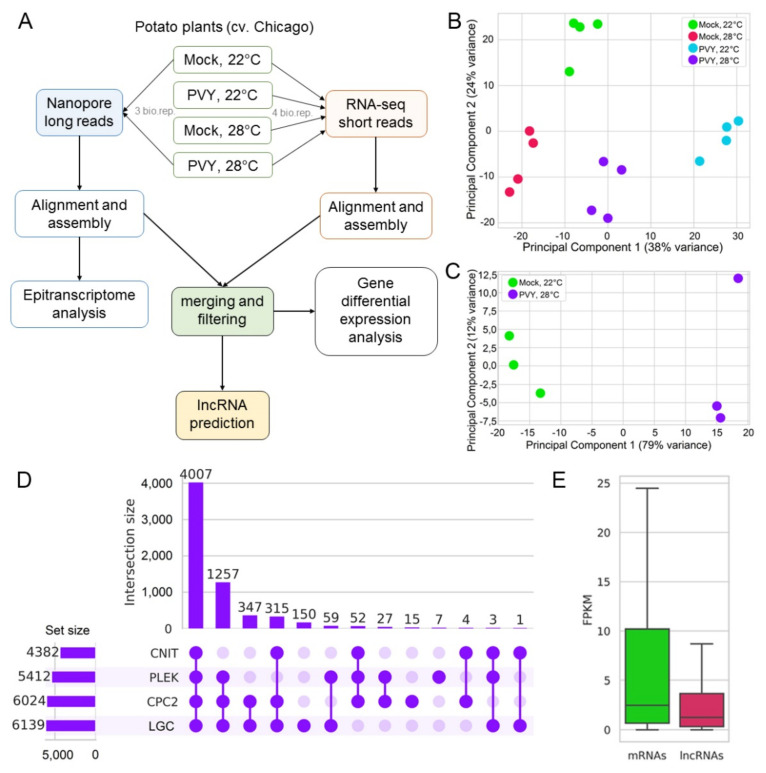
Overview of sequencing data. (**A**) Schematic representation of the experimental setup. (**B**) Principal Component Analysis (PCA) based on short-read sequencing. (**C**) Principal Component Analysis (PCA) based on long-read sequencing. (**D**) Prediction of lncRNA using 4 different models, CNIT (Coding-Non-Coding Identifying Tool), PLEK (Predictor of Long non-coding RNAs and mEssenger RNAs based on an improved K-mer scheme), CPC2 (Coding Potential Calculator) and LGC (ORF Length and GC content). (**E**) Expression of mRNAs vs. lncRNAs. FPKM, Fragments Per Kilobase of transcript per Million mapped reads.

To elucidate potential mechanisms of stress responses caused by PVY at a normal and elevated temperature, we performed a comprehensive analysis of potato transcriptomes, as illustrated in Figure 1A. Sixteen paired-end RNA-Seq libraries were generated from four biological replicates of mock-inoculated and PVY-infected plants at both normal (22 °C) and elevated temperatures (28 °C); sequencing was performed using the DNBSeq^TM^ technology platform (for short reads) (Figure 1A). In total 1,267,036,962 raw short reads were generated, from 75 to 100 million reads in every sample. More than 90% of the short reads were mapped to the annotated genome regions (Table 1). 

To gain insights into alternative splicing and to decipher epitranscriptomic changes, direct RNA nanopore sequencing was performed on three biological replicates for mock-inoculated plants at a normal temperature and PVY-infected plants at elevated temperature (combined stress conditions) (Table 2).

The RNA-seq short and long (nanopore) reads were assembled into 49,054 transcripts belonging to 26,942 loci (Table 3 and Table S1). About 40% of transcripts (18,842) exactly matched to those of the Phytozome V13 genome annotation. A further 23,989 transcripts were intersected with annotated genes but did not match them exactly. Thus, 87% of the detected loci were annotated and the transcriptome covered 69% of all annotated loci. Approximately 11% (5365) of transcripts belonged to novel loci and 2% of transcripts were antisense to known loci.

Additionally, we assembled transcripts from only the nanopore reads to make accurate annotations of alternative splicing events and to analyze epitranscriptomic modifications under combined stress conditions (PVY + heat) (Table S2). The final long-read dataset consisted of 46,488 transcripts from 25,252 loci (Table 3). The Principal Component Analysis (PCA) of short and long reads revealed significant differences between all samples obtained at various stress treatments (Figure 1B,C). Interestingly, the combined stress (PVY + heat) transcriptome has its own pattern, which is quite distinct from and does not overlap with other samples obtained under individual stresses (PVY or heat).

To annotate the assembled transcripts, the combined transcriptome was translated to 39,361 protein sequences containing more 100 amino acids (aa). The predicted proteins were annotated using InterProScan 5. Based on an E-cutoff value ≤ 1 × 10^−2^, the InterProScan analysis resulted in 98,282 matches to 8496 domain types. After InterProScan prediction 43,043 transcripts (87.7%) had annotated domains. The most represented domains in InterProscan included pentatricopeptide repeat domains, protein kinases, ribonuclease inhibitors, E-class P450 group I domains, leucine-rich repeat (LRR) containing regions and P-loop containing nucleoside triphosphate hydrolases.

Transcripts derived from 6245 loci which did not contain any ORFs longer than 100 aa or predicted domains were analyzed by 4 long non-coding RNA (lncRNA) annotation tools– CNIT (Coding-Non-Coding Identifying Tool), CPC2 (Coding Potential Calculator 2), PLEK (Predictor of Long non-coding RNAs and mEssenger RNAs based on an improved K-mer scheme) and LGC (ORF Length and GC content). Of them, 4007 transcripts were classified as noncoding by all models (Figure 1D). These transcripts were used for further analysis. Interestingly, 2785 lncRNAs (69.5%) were transcribed from unannotated loci, and only 83 fully matched annotated transcripts. It was also found that 317 lncRNAs were antisense to known genes. The expression of lncRNAs was significantly lower than mRNA expression (t = −3.337, *p* = 0.0008468997; Figure 1E).

### 2.3. Differential Expression of Genes under Stress Conditions

**Figure 2:**
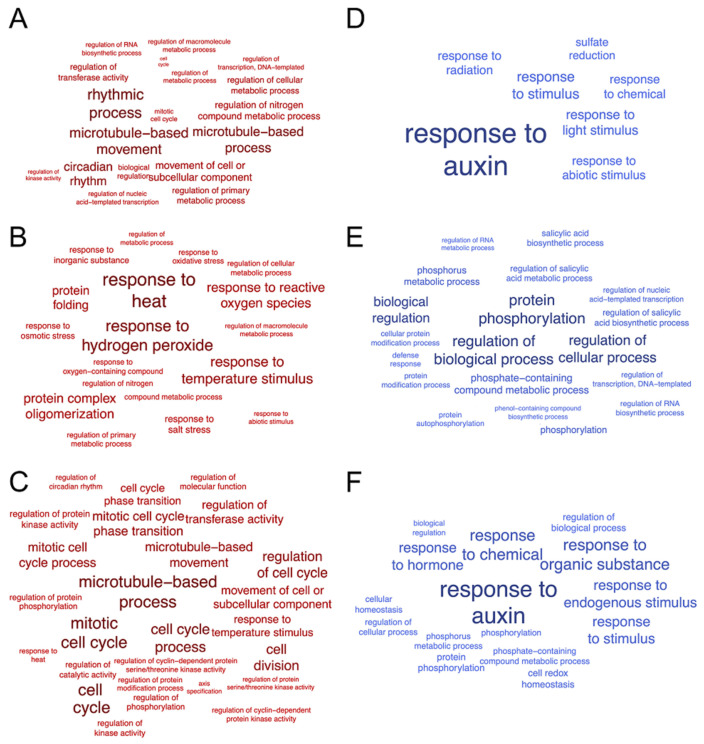
Gene ontology (GO) bias word clouds (biological process). Word clouds of genes upregulated and downregulated in mock-inoculated potato at 28 °C (**A**,**D**), PVY-infected potato at 22 °C (**B**,**E**) and 28 °C (**C**,**F**). Font size correlates with enrichment significance (see Table S4; g:Profiler software, *p* value < 0.05, corrected for multiple hypotheses testing by the Benjamini-Hochberg correction procedure [43]).

We used RNA-seq data to investigate the gene-expression patterns in potato at the elevated temperature, under PVY infection and combined stress conditions (PVY + heat), relative to uninfected plants at the lower temperature. The transcription levels of the assembled transcriptome were assessed using FPKM (Fragments Per Kilobase of exon per Million reads) values, and differentially expressed genes (DEGs) were defined based on an adjusted *p*-value <  0.05 and |log_2_ fold-change (log_2_FC)|  ≥  1. Based on these criteria, 1411, 2636 and 2342 DEGs were identified in mock-inoculated plants at 28 °C, under PVY infection at normal (22 °C) and under combined stress conditions, compared with control mock-inoculated plants at 22 °C, respectively (Table S3). Thus, virus infection at a normal temperature resulted in the most dramatic changes in potato transcriptome compared with other conditions, even with stress caused by PVY at the higher temperature. In plants infected at 22 °C, 1314 genes were down-regulated, and 1322 were up-regulated in comparison to mock-inoculated plants. Leucocyanidin oxygenase/leucoanthocyanidin dioxygenase (Soltu.DM.10G019650; FClog_2_ = 6.49), lysine-specific demethylase 8 (Soltu.DM.01G002360; FClog_2_ = 5.23) and lactoylglutathione lyase (Soltu.DM.01G042560; FClog_2_ = 5.16) were up-regulated to the greatest degree in infected plants. Among the most down-regulated genes were coniferyl-alcohol glucosyltransferase (Soltu.DM.02G006790; FClog_2_ = −7.39), hydroxycinnamate 4-beta-glucosyltransferase (Soltu.DM.02G006800; FClog_2_ = −5.97), ethylene-responsive transcription factor ERF019-related (Soltu.DM.01G017610, FClog_2_ = −5.5), heat shock transcription factors (Soltu.DM.09G020350, FClog_2_ = −4.57; Soltu.DM.09G020330, FC log_2_ = −4.41; Soltu.DM.09G020340, FC log_2_ = −4.36). An analysis of GO terms showed that microtubule-related terms (GO:0007018, GO:0007017, GO:0008017, GO:0003777) were enriched in up-regulated genes (Table S4, Figure 2), whereas the response to auxin (GO:0009733), response to light stimulus (GO:0009416), response to chemicals (GO:0042221) and response to abiotic stimulus (GO:0009628) were the most enriched categories in down-regulated DEGs relative to mock-inoculated plants at 22 °C (Figure 2). According to the KEGG pathway analysis, the down-regulated genes were enriched in a biosynthesis of secondary metabolites (KEGG:01110) and plant hormone signal transduction (KEGG:04075). Taken together, these data may suggest that PVY infection in virus-susceptible cultivar Chicago is associated with significant changes in general metabolic processes and hormone signalling, which may provide conditions for successful virus replication and spread throughout the plant.

There were fewer DEGs in plants grown under single heat stress than in PVY-infected plants. Relative to the mock-inoculated plants at 22 °C, mock inoculated plants at 28 °C had 1053 down-regulated and 358 up-regulated DEGs (Table S3). Among the most down-regulated DEGs, we identified aspartyl protease (Soltu.DM.01G024200, FClog_2_ = −9.14), glutaredoxins (e.g., Soltu.DM.04G007850, FClog_2_ = −5.69) and ethylene-responsive transcription factors, such as ERF019-related gene (Soltu.DM.01G017610.1 FClog_2_ = −7.77). The list of up-regulated DEGs included several members of the HSP family, such as small heat-shock protein HSP20 (Soltu.DM.03G021700, FC log_2_ = 3.29), MYB-like DNA-binding protein (Soltu.DM.06G034280, FClog_2_ = 2.52), Kunitz trypsin inhibitor gene (Soltu.DM.04G003450, FClog_2_ = 2.22) and transcription factor BHLH104-related protein (Soltu.DM.04G008320, FClog_2_ = 2.2). According to the GO enrichment analysis, down-regulated genes were mainly related to protein-phosphorylation processes (GO:0006468, GO:0006793, GO:0016310, GO:0046777, GO:0006796, Table S4, Figure 2). The KEGG pathways of down-regulated DEGs were also enriched in plant-pathogen interaction (KEGG:04626). With regard to up-regulated DEGs, they were assigned to biological processes such as responses to heat stresses (GO:0009408, GO:0009266) and responses to reactive oxygen species stimulus (GO:0042542, GO:0000302, GO:0006979; Figure 2). Thus, potato plants responded to the elevated temperature via an up-regulation of genes that mainly participated in heat stress responses, but some genes involved in plant–pathogen interaction were down-regulated. 

In our previous studies, we showed that the susceptibility to PVY was dramatically enhanced in Chicago plants under the elevated temperature [21,32]. To gain insight into plant response to the combined stress conditions (PVY + heat) at the transcriptomic level, we compared DEGs in control plants mock-inoculated at 22 °C and PVY-infected plants grown under the elevated temperature. At combined stress conditions, 1001 genes were up-regulated, and 1341 genes were down-regulated compared to the control plants (Table S3). Similar to heat stress conditions, among the most down-regulated genes were aspartyl proteases, glutaredoxins and ethylene-responsive transcription factors. In addition, wound-induced protein DUF3774 (Soltu.DM.02G018790, FC log_2_ = −3.70) was also strongly down-regulated at these conditions. According to the GO term analysis, down-regulated genes were enriched in biological processes such as responses to hormones (GO:0009733, GO:0009725) and endogenous stimuli (GO:0009719), and also phosphorylation (GO:0006468, GO:0016310, GO:0006793, Table S4; Figure 2). The KEGG pathways of down-regulated DEGs were enriched in plant–pathogen interactions (KEGG:04626) and plant hormone signal transduction (KEGG:04075). 

Among the most up-regulated DEGs at combined stress conditions (high temperature and PVY infection), gibberellin oxidase-like protein-related (Soltu.DM.06G032330, FC log_2_ = 4.3) and lysine-specific demethylase 8 (Soltu.DM.01G002360.1, FC log_2_ = 4.27) were identified. The up-regulated genes were enriched in cell-cycle-related processes (GO:0007049, GO:0000278, GO:0022402, GO:0051726, GO:1903047, GO:0044772), microtubule-related processes (GO:0007017, GO:0007018) and the regulation of various functions, including transferase activity (GO:0051338), protein kinase activity (GO:0045859) and some others (Figure 2).

In addition, to estimate the impact of the elevated temperature on plant responses to viral infection, we identified genes that were differentially regulated in mock- and PVY-infected plants both grown at 28 °C (Table S3). A group of DEGs which were down-regulated by the higher temperature in PVY-infected plants includes a number of various defensive proteins including LRR-containing proteins (e.g., Soltu.DM.06G011830, FLS2 LRR kinase), pathogenesis-related proteins such as ribonuclease T2 (Soltu.DM.05G005060), thaumatin (Soltu.DM.12G007860), wound-induced protein (DUF3774; Soltu.DM.07G019110) and heat-shock protein 2 (17.6 KDa HSP 2, Soltu.DM.09G001190). This group also composed membrane-channel proteins such as aquaporins (e.g., Soltu.DM.06G018020) and aquaporin transporters (e.g., Soltu.DM.03G012810). An increasing amount of evidence suggests that aquaporins play key roles in plant–pathogen interaction involved in plant immunity and pathogen pathogenicity [44]. The latter category of DEGs was enriched in GO terms such as water transport (GO:0006833) and fluid transport (GO:0042044) biological processes (Table S4). These observations indicating down-regulation of defensive proteins are in good agreement with the increased susceptibility of Chicago to PVY.

Another group of genes included DEGs that were up-regulated by the higher temperature in PVY-infected plants (Table S3). This up-regulated group, which includes heat-shock transcription factors (e.g., Soltu.DM.09G020350), was also found to be enriched in sulfate reduction (GO:0019419) and obsolete oxidation-reduction processes (GO:0055114, Table S4). The KEGG pathways of these genes were enriched in sulfur metabolism (KEGG:00920) and porphyrin and chlorophyll metabolism (KEGG:00860). Sulfur-containing compounds, especially glutathione, play vital roles in plant responses to stress conditions [45]. The functional relevance of the up-regulation of defensive sulfur-containing compounds and the increase in virus susceptibility in Chicago plants at higher temperatures is not clear. 

**Figure 3:**
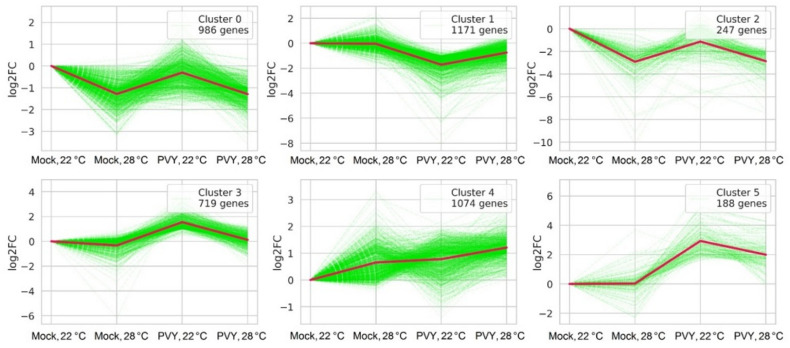
Differentially expressed genes: k-means clustering of DEGs based on log_2_FC values. Green lines represent individual genes, red lines mean log_2_FC cluster values.

To classify the genes with similar expression patterns under different conditions, k-means clustering was performed. Differentially expressed genes were divided into six clusters according to their expression patterns (Figure 3, Table S5). DEGs in clusters 0, 1 and 2 were mainly down-regulated and in clusters 3, 4, and 5 were mostly up-regulated in all three different stress conditions (PVY, heat, PVY + heat) compared with the control mock-inoculated plants.

The genes in clusters 0 and 2 were down-regulated during heat and combined stress conditions in comparison to control plants. In PVY-infected plants at 22 °C, both down- and up-regulated genes belonged to these clusters. For example, Cluster 0 contained endochitinase B (Soltu.DM.10G017910) and a member of alpha/beta-hydrolases superfamily (Soltu.DM.04G007210), both of which were significantly up-regulated in PVY-infected plants, but down-regulated in other stress conditions in comparison to the control. Cluster 2 included transcription factors (TFs) belonging to the MYB (e.g., Soltu.DM.03G027460) and WRKY (e.g., Soltu.DM.12G004050) families, which are one of the most important TF families in plants and play key roles in multiple plant-stress responses. (Table S5). According to the GO enrichment analysis, cluster 0 was enriched in biological processes such as protein phosphorylation (GO:0006468), response to auxin (GO:0009733) and response to stimuli (GO:0050896). The KEGG pathways were enriched in plant–pathogen interaction (KEGG:04626) and glutathione metabolism (KEGG:00480, Table S6). 

According to the GO terms analysis, DEGs from cluster 1 (which were down-regulated in all stress conditions, in particular in PVY-infected plants) were enriched in biological processes such as responses to hormones and various stimuli (e.g., GO:0009733, GO:0009725, GO:0042221, GO:0009628, GO:0050896, GO:0009416, Table S6). The KEGG pathways of cluster 1 were enriched in the biosynthesis of secondary metabolites (KEGG:01110, KEGG:00904), porphyrin and chlorophyll metabolism (KEGG:00860) and sulfur metabolism (KEGG:00920, Table S6). This cluster includes a group of different methyltransferases, such as S-adenosyl-L-methionine-dependent methyltransferase (Soltu.DM.01G026630), SAM-dependent O-methyltransferase class I-type enzymes (Soltu.DM.01G047320, Soltu.DM.12G013090), SAM-dependent O-methyltransferase class II-type enzymes (Soltu.DM.10G021480, Soltu.DM.08G001740) (Table S5). In addition to methyltransferases, S-adenosylmethionine synthase 2 gene (SAMS2) (Soltu.DM.12G001940) were found in cluster 1. As was shown previously, enzymes associated with methionine cycle and trans-methylation play pivotal roles in plant–virus interactions since they determine resistance or susceptibility to viruses [20,21,22]. 

The transcriptional rates of DEGs in Cluster 3 were clearly up-regulated during viral infection at 22 °C, but only changed slightly at 28 °C, regardless of the presence of the virus. The most up-regulated DEGs from this cluster included pectinesterase (Soltu.DM.09G023650), aquaporin (Soltu.DM.06G018020), cysteine-rich receptor-like protein kinase 2 (Soltu.DM.01G003480) and 1-aminocyclopropane-1-carboxylate oxidase 3 (Soltu.DM.12G023340), among others (Table S5). The latter gene is involved in the ethylene biosynthesis pathway, which is associated with the methionine cycle in plants. In addition, some methyltransferases, such as O-methyltransferases (e.g., Soltu.DM.02G016860) and serine hydroxymethyltransferase (SHMT) (Soltu.DM.01G006700) which participated in the methionine cycle pathways, were also identified in this cluster. 

The DEGs from Cluster 4 are mostly up-regulated under all stress treatments used in this work. In this cluster, we identified several members of the potato (proteinase) inhibitor I family (e.g., Soltu.DM.09G025900) that usually accumulate in response to mechanical damage [46]. However, some DEGs from this cluster were down-regulated during PVY infection at 22 °C, but up-regulated at 28 °C, with examples including ethylene-responsive transcription factor 13 (Soltu.DM.01G031000), ethylene-responsive transcription factor ERF035 (Soltu.DM.01G031210) and small heat-shock protein HSP20 (Soltu.DM.08G024980; Table S5). According to GO term, Cluster 4 was enriched in biological processes associated with microtubules and the cytoskeleton, phosphorylation and kinase activity, as well as responses to heat exposure (GO:0007049, GO:0007017, GO:0042325, GO:0009408; Table S6).

### 2.4. Functional Relevance of Differentially Expressed Protein-Coding RNAs

Similar to other viruses, PVY induces a tremendous remodeling of plant host (potato) transcriptomes [47,48,49]. Moreover, environmental conditions such as temperature contribute greatly to virus susceptibility/resistance and affect transcriptomes. Although seemingly simple relationships between changes in transcript expression and the modulation of downstream biological functions (and phenotypes) can sometimes be observed, this is quite often not the case. This obvious inconsistency is partly caused by technical difficulties and partly by the analytical problems associated with the complexity of biological processes and their poor elucidation. Technical problems may be caused, for example, by the fact that some transcripts may link to distinct protein IDs; on the other hand, some protein IDs may be linked to several transcripts which can have substantially different expression profiles (Table S1). With regard to analytical problems, many molecular mechanisms that underlie host physiological and phenotypic responses to virus infection and environmental stresses are still largely unknown (and poorly annotated). Therefore, changes in host gene expressions (activation or suppression) [47,50,51,52,53,54] are often difficult to unequivocally attribute to either processes that control stress responses (e.g., defence) or destructive effects of stresses on the plant.

However, the corollary of the data presented above is that the major gene-expression changes (both gene activation or suppression) caused by virus infection and/or elevated temperatures likely represent a combination of stress- and defence-related responses (such as pattern/effector triggered immunity, mitogen-activated kinase protein (MAP) cascades, hormone signal transduction, HSP-mediated-pathways and many others; Table S3), and the modulation of multiple metabolic processes (Table S3) which lead to plant-host disease development. Many of these processes may be regulated by transcription factors such as members of the MYB or WRKY families, which are regarded as key determinants for defence and development in plants and are affected by PVY infection and heat. Collectively, these observations are consistent with and confirm previously published data on transcriptomic and proteomic analyses of plant responses to virus infections and abiotic stresses [21,22,28,47,48,49].

However, this research also presents additional findings which do not lie in the mainstream of transcriptomic studies on plant responses to biotic and abiotic stresses, and are much less studies in the literature but may provide a good reference for future studies on molecular and cellular mechanisms induced in response to virus infections and possibly other kinds of stress.

**Figure 4:**
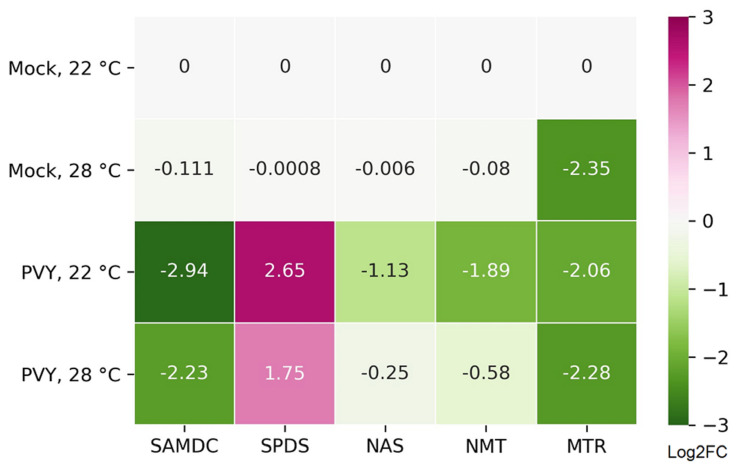
Heatmap showing the changes in abundance (log_2_FC) of mRNAs associated with the methionine cycle: adenosylmethionine decarboxylase (SAMDC; Soltu.DM.02G030190.1), spermidine synthase (SPDS; Soltu.DM. DM.06G014450.1), nicotianamine synthase (NAS; Soltu.DM.01G040240.1), phosphoethanolamine N-methyltransferase (NMT; Soltu.DM.12G011670.1) and 5-methyltetrahydrofolate-homocysteine methyltransferase (MTR; Soltu.DM.01G028040.1).

First, previous reports [20,21,22] suggest that antiviral defence mechanisms in plants may be regulated by MTC and its major function in trans-methylation processes. In particular, it has been shown that the enhanced susceptibility of Chicago plants to PVY at 28 °C may be orchestrated by the downregulation of MTC enzymes, which disturbs the MTC and its major function in trans-methylation processes [21]. This study extends the previous findings by predicting that additional pathways associated with MTC may also be involved in virus resistance (Figure 4). For example, polyamines such as spermidine and spermine are well known to be essential for various processes in plants, being implicated in many cellular functions including plant responses to abiotic and biotic stresses (such as virus attack) [55,56]. Interestingly the gene for adenosylmethionine decarboxylase (SAMDC; Soltu.DM.02G030190.1) an enzyme that plays an essential regulatory role in the polyamine biosynthetic pathway by the conversion of SAM to adenosylmethioninamine (decarboxylated SAM; DcSAM), is significantly downregulated by all the treatments used in this work (Table S3; Figure 4). At the same time, the gene for spermidine synthase (SPDS; Soltu.DM.06G014450.1) which catalyses the transfer of the propylamine group from DcSAM to putrescine in the biosynthesis of spermidine [55] was upregulated in response to PVY infection at both the normal and higher temperature (Figure 4). Moreover, genes encoding nicotianamine synthase (NAS, Soltu.DM.01G040240.1) and phosphoethanolamine N-methyltransferase (NMT, Soltu.DM.12G011670.1), which also used SAM as a substrate or donor, respectively, were downregulated in response to PVY (Figure 4). Thus, all these processes involving SAM are potentially competing with each other, and their balance may serve as check points for important plant defence responses such as trans-methylation, biosynthesis of polyamines and ethylene. A dramatic reduction in their expression may lead to enhanced susceptibility to PVY [20,21,22,55,56]. Consistent with this, the expression of 5-methyltetrahydrofolate-homocysteine methyltransferase (MTR, methionine synthase; Soltu.DM.01G028040.1) converting homocysteine into methionine (an immediate precursor of SAM) was significantly downregulated (Table S3; Figure 4).

**Figure 5:**
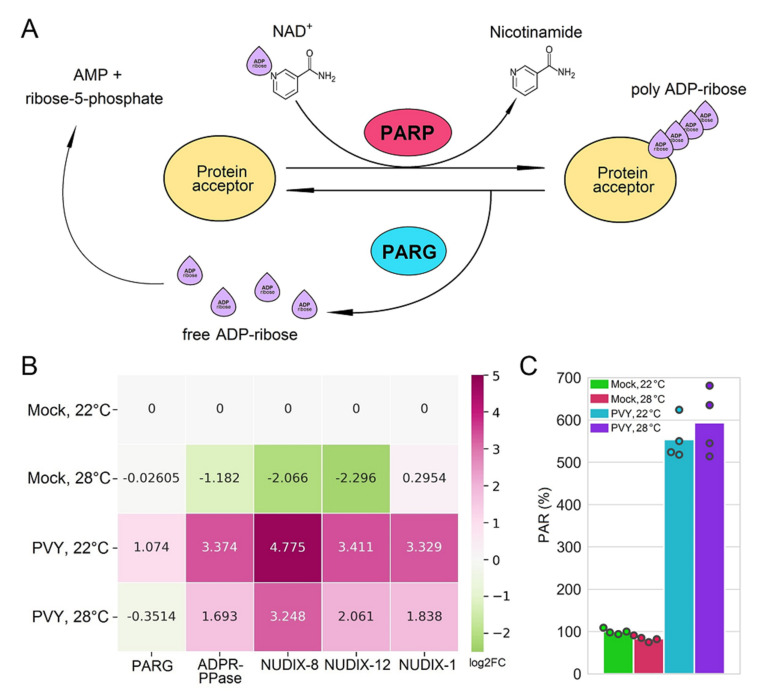
Poly (ADP-ribose) metabolism and susceptibility of Chicago plants to PVY. (**A**) Schematic representation PARylation process in healthy cells. Poly(ADP-ribose) polymerase (PARP), poly(ADP-ribose) glycohydrolase (PARG), and nucleotide diphosphate linked to some moiety-X (NUDIX) enzymes. PARP enzymes bind NAD+ (nicotinamide adenine dinucleotide), cleave off the nicotinamide residue, and attach the remaining ADP-ribose moiety to acceptor proteins (protein X, which can include PARP itself). PARG then cleaves the ribose–ribose backbone bond of poly(ADP-ribose), releasing free ADP-ribose. ADP-ribose-specific NUDIX enzymes then cleave free ADP-ribose into AMP (adenosine monophosphate) and ribose-5-phosphate. (**B**) Heatmap showing the changes in abundance (log_2_FC) of mRNAs related to the poly ADP-ribosylation process: PARG (Soltu.DM.12G003820.1), ADPR-PPase (ADP-ribose pyrophosphatases; Soltu.DM.08G000850.1) and NUDIX enzymes (Soltu.DM.03G005230.1-NUDIX-1; Soltu.DM.08G000940.1—NUDIX-12; Soltu.DM.08G000920.1-NUDIX-8). (**C**) Accumulation of PARylated proteins measured by ELISA using rabbit anti-PAR polyclonal antibody, in PVY- systemically infected or mock-inoculated plants at 22 °C or 28 °C. Analysis of variance and Tukey’s HSD post hoc tests were performed for data.

Second, we observed significant changes in the expression of genes that regulate the post-translational modification process by which polymers of ADP-ribose [poly(adenosinediphosphate-ribose), PAR] are covalently attached to proteins by PAR polymerase enzymes (Figure 5). The central enzyme for PAR production in cells and the main target of PARylation is poly(ADP-ribose) polymerase 1 (PARP1). Interestingly, PARP1 modifies the function of a variety of nuclear “target” proteins by attaching chains of ADP ribose (PAR) to them and itself [57,58,59]. To re-activate these target proteins, PARP1 shuttles them from the nucleolus and chromatin to Cajal bodies (CBs; prominent subnuclear compartments) for PAR removal and recycling by poly(ADP-ribose) glycohydrolase (PARG) [60,61,62]. The further degradation of free ADP-ribose into AMP and ribose-5-phosphate occurs as a result of nucleoside diphosphate linked to some moiety-X (NUDIX) hydrolases with specificity for ADP-ribose or ADPR-PPase (ADP-ribose pyrophosphatases; [57]. It is well known that in plants, as in animals, PARylation plays an important role in various biological processes, including responses to biotic and abiotic stresses [57,58,59]; however, the molecular mechanisms and proteins involved are largely unknown. 

Interestingly, we observed a consistent and concerted upregulation of various NUDIX enzymes (NUDIX-1, Soltu.DM.03G005230.1; NUDIX-8, Soltu.DM.08G000920.1; NUDIX-12; Soltu.DM.08G000940.1.) and ADP-PPase (Soltu.DM.08G000850.1) in response to PVY infection at both the normal and higher temperature (Figure 5A,B). The gene for PARG (Soltu.DM.12G003820.1) was also upregulated in response to PVY at the normal temperature. Probably because of these changes, PAR accumulation to was significantly increased in Chicago plants infected with PVY compared to non-infected plants (Figure 5C). We hypothesise that the over-accumulation of PAR induced by PVY infection may play an important role in responses of Chicago plants to PVY, although a correlation between virus loads (at high and normal temperatures) and levels of PARylation was not observed (Figure 5C), which may be explained by the negative effect imposed by high temperature on the expression of *PARG*, *NUDIX* and *ADPR-PPase* (Figure 5C). It is thus intriguing to speculate on whether the PVY–plant interactions are at least partially regulated by PARylation functions, and also to what extent this mechanism could control plant responses to other biotic and abiotic stresses [59]; potential pathways which warrant future investigation. 

### 2.5. PVY Accumulation and Validation of Differentially Expressed Genes by qRT-PCR

**Figure 6:**
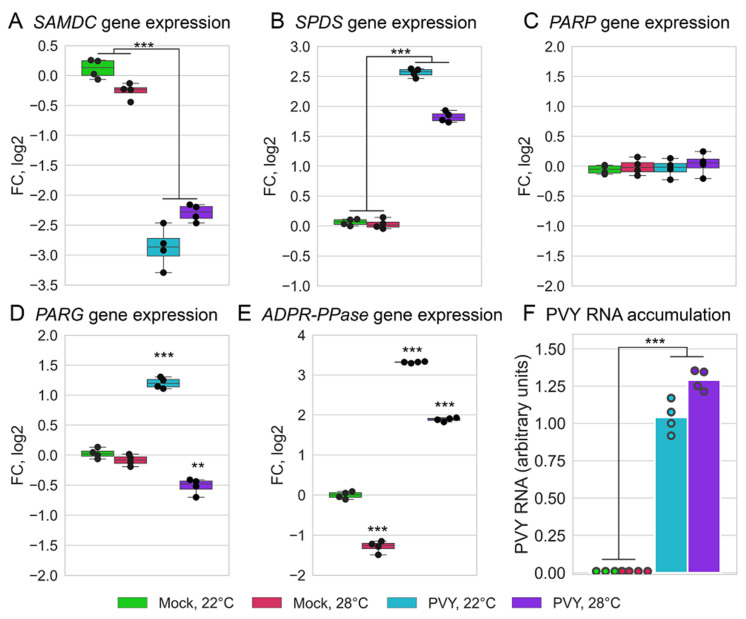
The dynamic expression patterns of important genes associated with MTC (*SAMSDC, SPDS*) and PARylation processes (*PARP, PARG* and *ADPR-PPase)* (**A**–**E**) and PVY RNA accumulation (**F**) analysed by RT-qPCR in systemically infected leaves of Chicago plants at 22 or 28 °C at 8 dpi, as shown. SAMDC, adenosylmethionine decarboxylase (Soltu.DM.02G030190.1); SPDS, spermidine synthase (Soltu.DM. DM.06G014450.1); PARG, poly(ADP-ribose) glycohydrolase (Soltu.DM.12G003820.1); PARP, Poly(ADP-ribose) polymerase (Soltu.DM.03G032200.1); ADPR-PPase, ADP-ribose pyrophosphatases (Soltu.DM.08G000850.1). Analysis of variance and Tukey’s HSD post hoc tests were performed on the RT-qPCR data. ** *p* < 0.01, *** *p* < 0.001.

Viral RNA accumulation was verified in non-inoculated (systemically infected) leaves of PVY-infected plants grown at normal (22 °C) and elevated (28 °C) temperatures via a RT-qPCR analysis of total RNA at 8 dpi, a time point at which samples for RNAseq analysis were also collected. PVY-accumulation rates were essentially similar at both these temperatures at this early stage of infection (Figure 6F), however a significant rise in PVY RNA levels was detected at 28 °C at the later stages [21,32]. 

It should be noted that when the polyadenylated RNA was analyzed, short-read sequencing revealed only 10,048 virus-specific reads, equating to 0.003% of the total reads (306,078,702) obtained at 28 °C (20.3 FPKM), whereas the number of viral reads at 22 °C was much lower (89 reads per 295,131,924 total reads; 0.07 FPKM). The reasons for this inconsistency remain unclear, but a possible contributor is that although PVY RNA is typically polyadenylated [2], virus abundance may be underestimated, for example, because of the possibly insufficient polyadenylation of viral RNA at early stages of the infection cycle, especially at 22 °C.

To validate the RNAseq data, the transcriptional expressions of four differentially expressed genes, randomly selected from those associated with the MTC (*SAMDC*, *SPDS)* and PARylation process (*PARG, ADPR-PPase)*, and one non-regulated gene (*PARP1*) were further analyzed by RT-qPCR (Figure 6A–E). The expression trends of these five genes by RT-qPCR were highly consistent with those from RNAseq analyses (Figure 6A–E vs. Figure 4 and Figure 5B). 

### 2.6. Differential Expression of LncRNAs under Stress Conditions

**Figure 7:**
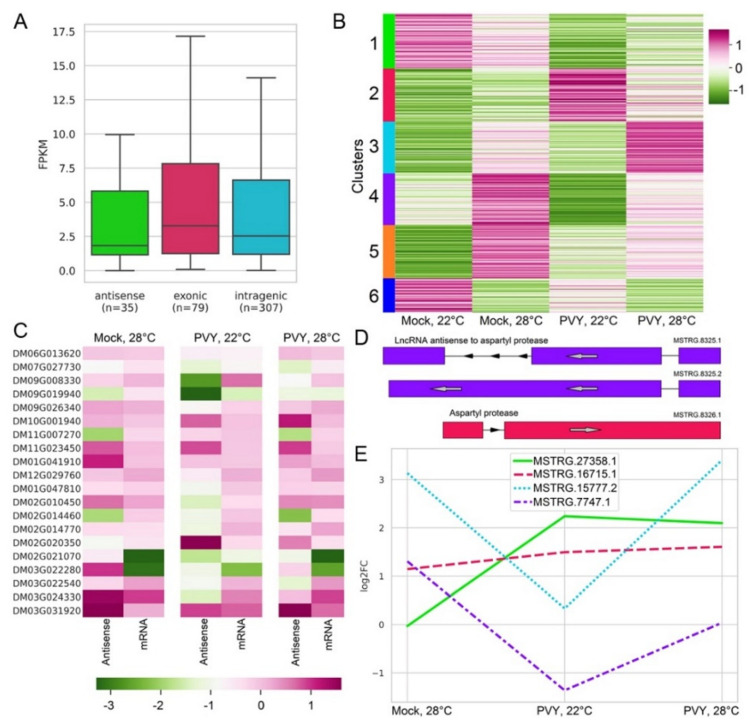
Differential expression of long non-coding RNAs. (**A**) Distributions of transcript expression of mRNAs and lncRNAs. (**B**) Heatmap and k-means clustering based on FPKM values of differentially expressed lncRNAs. (**C**) Heatmap comparing log_2_FC of lncRNAs antisense to mRNAs and corresponding mRNAs. (**D**) An example of StringTie annotation of antisense lncRNA and corresponding mRNA which were up-regulated under PVY infection. (**E**) Log_2_FC of chosen lncRNAs with miRNA target sites.

It has recently been shown that plant lncRNAs can play regulatory roles in plant responses to stress conditions [63]. To provide new insights into the roles of noncoding transcripts in stress responses in potato, we identified 421 differently regulated lncRNAs (DE-lncRNAs) that significantly changed (*P*adj < 0.05) their transcriptional level under at least one stress condition (Table S7). These DE-lncRNAs were classified into three groups—intergenic (*n* = 307), exonic or intronic (*n* = 79), and antisense transcripts (*n* = 35) (Figure 7A). The six different expression patterns of DE-lncRNAs were identified across three stress conditions (Figure 7B). We found four DE-lncRNA clusters that were up-regulated under PVY infection (cluster 2), elevated temperature (clusters 4 and 5) and combined stress (PVY + heat) conditions (cluster 3). However, we did not identify any DE-lncRNAs that were up-regulated in all stress conditions, suggesting a condition-specific regulation of this type of transcript. In particular, the 75 DE-lncRNAs that belonged to Cluster 2 were significantly up-regulated in infected plants infected with PVY at 22 °C, but most of them returned to normal under rising temperature (28 °C) (Figure 7B). Some lncRNAs, such as intergenic transcripts MSTRG.22592.2 and MSTRG.13078.1, were significantly up-regulated in PVY-infected plants at the normal temperature, but down-regulated during infection at the elevated temperature (Figure 7B). These findings confirm the idea that responses to viruses are strongly modulated by temperature.

We identified 221 DE-lncRNAs from Clusters 3, 4 and 5 which were up-regulated under the elevated temperature regardless the presence of PVY, suggesting that these DE-lncRNAs could belong to temperature-responsive lncRNA transcripts in potato. Some of these transcripts (27 from 77 in cluster 3) were significantly up-regulated at an elevated temperature, but down-regulated during infection at the normal temperature (Figure 7B). In addition, we identified two clusters (1 and 6) that were downregulated under stress conditions. As such, transcripts from cluster 1 (77 lncRNAs) were downregulated during PVY infection, and 48 lncRNAs from Cluster 6 were down-regulated under higher temperature and combined stress (PVY + heat) conditions (Figure 7B).

It has been previously reported that antisense lncRNAs are involved in gene-expression regulation under various stress conditions [63]. Therefore, we analysed the pair sense–antisense transcripts to determine whether the antisense DE-lncRNAs can regulate stress-related genes. In total, we identified 22 antisense DE-lncRNAs, but only a few of them changed in a coordinated manner with the corresponding mRNA (Figure 7C, Table S8). For example, an antisense DE-lncRNA MSTRG.5049 and the annotated gene of wall-associated receptor kinase galacturonan-binding (GUB_WAK_bind) protein (Soltu.DM.02G021070.1) were both significantly down-regulated in PVY infected plants. Furthermore, 3-exon lncRNA MSTRG.8325 was antisense to aspartyl protease Soltu.DM.03G031920 (Figure 7D). The protease and antisense transcript were up-regulated under PVY infection at both normal and high temperatures. We also identified an example of opposite regulation under the single heat stress. The up-regulation of antisense lncRNA MSTRG.7580.1 resulted in the down-regulation of the corresponding gene Soltu.DM.03G022280 from the alpha/beta hydrolase (ABH) superfamily. Interestingly, ABH enzymes serve as the core structure for phytohormone and ligand receptors in various signaling pathways in plants [64].

It has been previously shown that lncRNAs can act as decoys, limiting the availability of different regulatory factors, such as miRNAs [65]. To gain insight into the role of such interactions in stress responses, we then searched for lncRNAs with possible binding sites on miRNAs previously identified in potato [66,67]. Overall, we identified 139 lncRNAs (90 intergenic and 16 antisense) bearing 275 miRNA binding sites (Table S9). Of them, 21 DE-lncRNAs with predicted miRNA target sites were detected. We then selected miRNAs that are known to participate in stress response regulation in potato, based on the data from [66]. For example, binding sites for miR530 (MSTRG.15777.2), a known regulator of immune responses in plants [68] were found on 3 DE-lncRNAs. Two of these transcripts were upregulated under single heat stress (Table S9).

Another example of miRNA involved in regulating many processes, including heat-stress response, is miR390. Previously, MiR390 was shown to take part in the regulation of auxin response factors ARF3/4 [69]. The lncRNA decoy (MSTRG.16715.1) (Figure 7E) that potentially interacts with miR390 showed upregulation under all stress conditions.

Additionally, A lncRNA MSTRG.27358.1, that contains binding sites for miR396, was up-regulated under the elevated temperature in the presence or absence of the virus (Figure 7E). The miR396–GROWTH REGULATING FACTOR (GRF) class-GRF-INTERACTING FACTOR (GIF) network is an important module affecting plant growth. Additionally, MiR396 is an evolutionarily conserved miRNA which was shown to repress GRF genes [70,71]. Up-regulation of miR396 under stresses inhibits plant growth through the suppression of the GRF in Arabidopsis [71]. 

A further fifteen lncRNAs with predicted binding sites for potato specific miRNA were down-regulated under at least one of the stress conditions. For example, a lncRNA MSTRG.7747.1 has a predicted binding site for miRNA102 and was up-regulated under the elevated temperature (in the presence or absence PVY), but down-regulated under PVY infection at 22 °C (Figure 7E). This lncRNA is antisense to a mitochondrial 28S ribosomal protein S21 (Soltu.DM.03G024330.1), which is up-regulated under single heat and combined (heat + PVY) stress (Table S8).

### 2.7. Differentially Expressed Isoforms: Alternative Splicing

**Figure 8:**
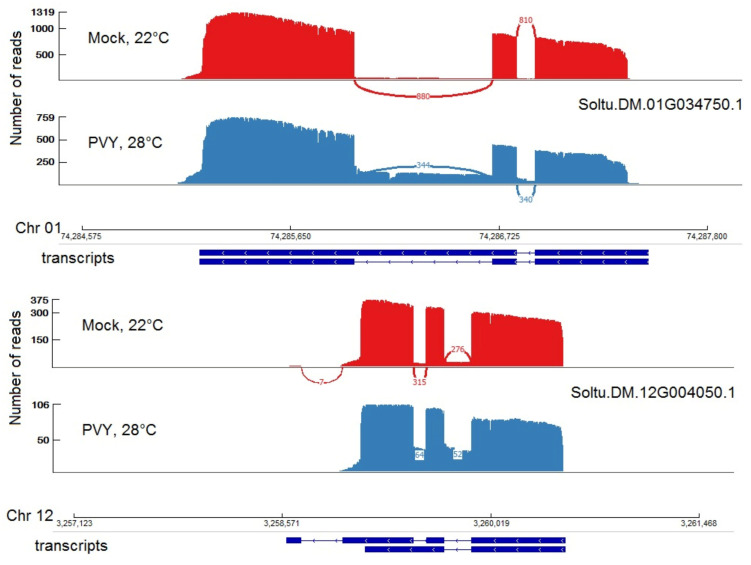
Sashimi plots of Nanopore data demonstrating examples of two WRKY transcription factors with upregulation of isoform with intron retention in plants under combined (PVY + heat) stress. The main panel shows counts of the reads that span the junctions. The transcripts correspond to chr01:74285180..74287496 and chr12:3259116..3260538 according to chromosome (chr) positions of Phytozome database *Solanum tuberosum* genome V6.1.

Alternative splicing (AS) is a mechanism responsible for the generation of numerous protein isoforms from a single gene. AS plays an important role in plant stress responses, however, the correct detection and quantification of isoforms is a challenging task. Using the advantages of nanopore sequencing to currently identify full-length transcripts, we analyzed differentially spliced potato genes in response to combined stress (PVY + heat) treatment. For this, the transcriptome assembled from nanopore reads was used. We compared the proportion of each isoform in the level of gene expression using the Fisher exact test. Overall, 8705 genes with two or more isoforms were analyzed. We identified 283 transcripts from 176 loci that showed significantly different proportions between mock-inoculated plants and plants infected with PVY under elevated temperature (Table S10). Twenty-six isoforms are associated with genes encoding WRKY transcription factors that play an important role in plant tolerance to biotic and abiotic stresses [72]. Examples of two such genes are illustrated in Figure 8. Both of them had significantly up-regulated intron-retained isoforms in the stressed plants.

### 2.8. Epitranscriptome Analysis: m^6^A RNA Methylation

RNA modifications are an important regulator of numerous cell processes [73,74]. Among the diverse modifications found for mRNAs, N6-methyladenosine (m^6^A) is the most prevalent modification in both plants and animals [75]. Additionally, m^6^A RNA methylation may be pivotal for posttranscriptional gene-regulatory events such as mRNA splicing, stability, and translation. The ability to control the fate of RNA molecules through nucleotide modifications is vital to plant health and survival under diverse environmental conditions. To provide more insights into the role of RNA methylation in the stress responses of potato we used direct RNA nanopore sequencing, which provides an opportunity to predict sites of RNA methylation. Here, we used the xPore tool for analysis of the differences in m^6^A RNA methylation between control mock-inoculated plants maintained at 22 °C and plants infected with PVY at the higher temperature. Overall, 57 transcripts might be differentially modified, some of which are involved in responses to stress conditions (Table S11). Most of the predicted differentially modified positions were located in RNA sites corresponding to coding DNA sequences (CDS; 68%) and 3′-untranslated regions (UTRs; 19%). Only two differentially modified transcripts belonged to DEGs. For example, alternative splicing factor SRp20/9G8 (Soltu.DM.12G000050; RRM superfamily) had predicted methylation sites with a higher modification rate in stressed plants in comparison to control (Table S11). Interestingly, SRp20/9G8 belongs to a family of serine and arginine-rich (SR) proteins that are key determinants of exon identity, and function as molecular adaptors, linking the pre-mRNA to the splicing machinery [76].

Another example is the ABH superfamily protein (Soltu.DM.07G000230) that has a higher methylation rate for sites located in the 3′-UTR region (Differential modification rate = 34%) of the transcript (Table S11). This gene was up-regulated in PVY-infected plants at 22 °C, but down-regulated in other stress conditions. The ABH superfamily includes a wide range of enzymes that have diverse functions, such as esterases, lipases, thioesterases, amidases, epoxide hydrolases, dehalogenases, haloperoxidases, and hydroxynytrile lyases. Many of them may operate in adaptations and responses to a wide range of biotic and abiotic stresses to combat the deleterious effect on their survival [63]. 

Intriguingly, in addition to mRNAs, we also predicted methylation events on PVY RNA in two of the three biological replicates from plants infected with PVY at 28 °C. Since PVY RNA was obviously absent in the mock-inoculated samples, we could not use the xPore tool for an analysis of differently methylated transcripts. Therefore, we used the m6Anet software for the prediction of methylation events. The M6Anet tool showed 2033 modified positions, 78 of which were located on the virus sequence (Table S11). The highest putative methylation rates (>86%) were identified for positions 6806 and 9583 on PVY transcript (NC_001616). They were located in the NIa-Pro protein and 3′-UTR region, respectively. 

Previous studies have shown that the m^6^A machinery methylates the viral RNA genomes of several animal and plant viruses [77,78] including cucumber mosaic virus and alfalfa mosaic virus, although the functional role of such methylation remains obscure. The biological significance of PVY RNA methylation as well as host plant mRNA methylation in response to PVY infection and other biotic and abiotic stresses is also not known and will be addressed in future research.

## 3. Materials and Methods

### 3.1. Plant Material

To infect potato plants, an ordinary (O) strain of PVY (PVY^O^) was used [79]. The virus was maintained in fresh plant material (*Nicotiana bentamiana*), which was then extracted in a potassium phosphate (KP) buffer pH 7.5 (1:3 w/v). Four-week-old potato plants (*Solanum tuberosum* L.; cv. Chicago) were inoculated with 200 µL PVY extract; control plants were mock-inoculated with KP buffer. Plants were kept in a controlled environment chamber (Pol-Eko-Aparatura, Wodzisław Śląski, Poland) with a photoperiod of 16/8 h day/night at a relative humidity of 50% and a temperature of 22 °C, with a light fluence of 250 µmol m^−2^·s^−1^. At 2 dpi (days post-inoculation), half of the plants were transferred to 28 °C for heat-stress simulation. Leaf samples from four virus-inoculated (systemically infected leaves) or control mock-inoculated plants were collected at 8 dpi. From each plant, 3–4 leaves were collected and pooled together. The harvested material was processed and analysed as described below.

### 3.2. RNA Isolation, Library Preparation, and Sequencing

Total RNA was isolated from the liquid-nitrogen-frozen and ground up leaf tissue using the Invitrogen™ TRIzol™ Reagent (ThermoFisher Scientific™, USA), following the manufacturer recommendations. The precise concentration of total RNA in each sample was measured using a Quant-iT™ RNA Assay Kit in conjunction with a Qubit 3.0 fluorometer (Invitrogen, US), with a standard curve range of 20–1000 ng of RNA. A total of 100 μg aliquots of total RNA was diluted in 100 μL of nuclease-free water and poly(A) containing RNA was isolated using and following the recommendations of the Thermo Fisher Scientific Poly(A)Purist™ MAG Purification Kit Invitrogen. The resulting poly(A) RNA was eluted in nuclease-free water. Paired-end RNA-Seq libraries were generated from the poly(A) RNA isolated from mock-inoculated and PVY-infected plants; short read sequencing was performed using the DNBSeq^TM^ technology platform. The Direct RNA sequencing kit from Oxford Nanopore (SQK-RNA002) including the optional reverse transcription step was used to prepare libraries from thepoly(A) RNA. Total library sequencing was performed on 200 ng of each poly(A) RNA library using the MinION platform in conjunction with FLO-MIN106 (ONT R9.4) flow cells and the standard MinKNOW software. All obtained reads were deposited in the NCBI database; BioProject accession PRJNA786109.

### 3.3. Alignment to Genome and Transcriptome Assembly

We used Guppy 4.0.15 (Oxford Nanopore Technologies) for basecalling direct RNA sequencing data. MinIONQC was used for quality control [80]. Reads were aligned to the *S. tuberosum* genome V6.1 [81] with added *S. tuberosum* plastid and mitochondrial sequences, and *Saccharomyces cerevisiae* enolase control RNA sequence. Minimap2.20 [82] with parameters -ax splice -uf -k14 f -G2k --secondary = yes was used for the alignment. Samtools 1.9 [83] and Bedtools2.27.1 [84] were used to retrieve sorted bam files and alignment statistics. Short paired-endisat2 [85] and StringTie2 [86] were used for genome-guided transcriptome assembly. The parameters used were as follows: -L -B for each sample of long reads, default parameters for short reads and --merge -i -g5 for merging. To find intersection types between assembled transcriptome and other annotations we used GffCompare [87].

### 3.4. Differential Expression Analysis

The FeatureCounts [88] tool was used for short-read quantification to find DEGs. NanoCount [89] was used for long-read quantification and kallisto [90] for short read quantification to transcripts. FPKM values were counted with Picard [91] and R package countToFPKM [92]. Differential expression analysis was performed using DESeq2 [93] and apeglm [94] R packages with a cutoff of 10 reads for each feature. DESeq2 was also used to build PCA (Principal Component Analysis). Clusters were predicted with the scikit-learn Python package [95] using the k-means algorithm. 

### 3.5. GO Analysis

To find the enrichment of GO terms g:Profiler [96] was used. The R package tag cloud was used to build word cloud [97].

### 3.6. Functional Annotation

Open reading frames with lengths >30 a.a. were predicted in assembled transcriptome with Python package orfipy. These ORFs were used for domain prediction with InterProScan [98]. Loci without any transcripts with predicted ORFs with lengths >100 a.a or predicted domains on them were used for coding a potential prediction. Before the prediction, transcripts with hits on the Rfam database (E-value < 0.01) [99] were discarded. Homology to Rfam was detected with the Infernal pipeline [100]. LncRNAs were predicted using the following four models: CNIT [101] with option ‘plant’, PLEK [102] and two models CPC2 and LGC as a part of ezLncPred toolbox [103]. UpSet plot was built in UpSetplot 0.5.0 Python package [104]. Transcripts predicted as non-coding by all models were considered as lncRNAs. MiRNA target sites were predicted with the TAPIR [105] web tool.

### 3.7. Isoform Expression and Epitranscriptome Analysis

Isoform ratios were counted based on long reads TPM values. Differences in the ratios between mock-inoculated (22 °C) and PVY-infected (28 °C) plants were counted using Fisher exact test. RNA modifications were predicted with m6Anet [106] and xPore [107], using default parameters. The *p*-values of xPore and isoform ratio differences were adjusted using Benjamini–Hochberg procedure. Sashimi plots were built in the Integrative Genomics Viewer (IGV) [108].

### 3.8. Immunological Detection of Poly ADP-Ribose (PAR)

For the isolation of plant nuclei and nuclear protein extraction, the CelLytic PN kit (Sigma Aldrich) was used [109]. The protein (5 μg) was analysed for PAR accumulation levels by ELISA using (Trevigen) purified monoclonal antibody to PAR as the capture reagent, a rabbit anti-PAR antibody (Trevigen) as the detecting agent, and a goat anti-rabbit antibody conjugated with alkaline phosphatase (Sigma Aldrich) as the reporter [110]. PAR polymer (Trevigen) was used as a positive control.

### 3.9. RTqPCR

Total RNA was isolated as described above. DNase-treated RNA was reverse-transcribed into cDNA using the SuperScriptTM First-Strand Synthesis System for RT-PCR (Invitrogen), with either an oligo-dT primer (for host plant-specific mRNAs) or a PVY-specific primer (see Table S12). The primer pairs for SYBR green-based real-time PCR analysis of PVY RNA and host mRNAs (which are listed in Table S12) were designed using both Plant Genomics Resource Phytozome (https://phytozome.jgi.doe.gov/pz/portal.html (accessed on 26 January 2022)) and PRIMER EXPRESS software (ThermoFisher Scientific, Waltham, MA, USA). The Ct values for PVY RNA and each mRNA of interest were normalized using two internal reference genes encoding StEF-1α [111] and cytochrome c oxidase subunit 1 (StCOX) [48]. The average Ct values of the two reference genes was used to analyse PVY and host mRNA levels.

## 4. Conclusions

We have previously shown that the enhanced susceptibility of Chicago potato plants to PVY at high temperatures may be triggered by MTC perturbation and the resultant reduction in transmethylation activities [21,22]. The work we currently present extends these findings by identifying and predicting additional pathways associated with the MTC, which may also be involved in virus resistance (Figure 4). In particular, polyamines such as spermidine and spermine are well known to be essential for various processes in plants, being implicated in many cellular functions including plant responses to abiotic and biotic stresses (such as virus attack). The gene for adenosylmethionine decarboxylase, an enzyme that plays an essential regulatory role in the polyamine biosynthetic pathway by the conversion of SAM to DcSAM, was significantly down-regulated upon all the treatments used in this work. At the same time, the gene for spermidine synthase, which catalyses the transfer of propylamine group from DcSAM to putrescine in the biosynthesis of spermidine, was up-regulated in response to PVY infection at both the normal and higher temperature. Thus, resistance to PVY may be regulated by various pathways related to MTC.

Another novel mechanism of high susceptibility of Chicago plants to PVY may be associated with the over-accumulation of PARylated proteins in the PVY-infected leaves, which is supported by the negative effect of high temperature on the expression of *PARG*, *NUDIX* and *ADPR-PPase* (Figure 5). It is thus intriguing to speculate on whether the PVY–potato plant interactions are at least partially instigated by PARylation function.

Single and combined (PVY and heat) stress treatments also cause dramatic changes in the expression of many lncRNAs, RNA methylation and alternative splicing. In our analyses, we were able to recover some known regulators and pathways in the responses to virus infections and abiotic stress as well as some putative novel regulators and pathways, which provide new insights for understanding the mechanisms of plant stress tolerance. Collectively, these data demonstrate that plant responses to biotic (virus) and abiotic (heat) stresses are highly complex and involve changes at many levels. Moreover, plants respond to combined (multiple) stresses differently from single stresses, completely reprogramming the gene-expression pattern corresponding to the particular environmental conditions. These interactions between biotic and abiotic stresses are concerted by hormone-signalling pathways and changes in gene expression, including coding and non-coding RNAs transcription, alternative splicing and epitranscriptomic and epigenetic events that act together in a complex integrated network. The identification of master regulators that integrate all these mechanisms is therefore crucial for the development of new approaches applicable for the improvement of broad-spectrum stress-resistance in crops.

## Figures and Tables

**Table 1:** Short reads sequencing statistics.

Identification	NCBI Accession	Number of Reads	Number of Read Pairs	Q20 (%)	Aligned to the Genome (%)
Mock, 22 °C_1	SRR17129393	72,472,982	108,709,473	97.86	93.20
Mock, 22 °C_2	SRR17129392	72,465,906	108,698,859	97.66	93.45
Mock, 22 °C_3	SRR17129381	75,141,894	112,712,841	97.57	92.96
Mock, 22 °C_4	SRR17129378	95,142,250	142,713,375	97.84	92.24
Mock, 28 °C_1	SRR17129377	75,125,564	112,688,346	97.78	93.08
Mock, 28 °C_2	SRR17129376	100,155,380	150,233,070	97.71	91.76
Mock, 28 °C_3	SRR17129375	89,289,122	133,933,683	98.09	92.25
Mock, 28 °C_4	SRR17129374	86,033,238	129,049,857	97.70	91.88
PVY, 22 °C_1	SRR17129373	72,632,520	108,948,780	97.79	93.21
PVY, 22 °C_2	SRR17129372	72,454,368	108,681,552	97.96	93.33
PVY, 22 °C_3	SRR17129391	74,958,594	112,437,891	97.77	92.88
PVY, 22 °C_4	SRR17129390	75,086,442	112,629,663	97.71	93.02
PVY, 28 °C_1	SRR17129389	83,177,956	124,766,934	97.83	91.46
PVY, 28 °C_2	SRR17129388	75,185,626	112,778,439	97.67	92.66
PVY, 28 °C_3	SRR17129387	72,652,314	108,978,471	97.90	93.21
PVY, 28 °C_4	SRR17129386	75,062,806	112,594,209	97.60	92.51

Q20 (%)-percent of reads with Pherd quality score higher than 20. Aligned to the genome (%)-percent of reads mapped to the *Solanum tuberosum* genome reference sequence.

**Table 2:** Nanopore sequencing statistics for reads Q > 7.

Identification	NCBI Accession	Total Reads	Reads N50	Median Q Score	Median Length (bp)	Aligned to the Genome (%)
PVY 28 °C_1	SRR17129382	2,912,102	1038	10.5	796	99.07
PVY 28 °C_2	SRR17129380	2,231,219	1089	9.9	838	99.2
PVY 28 °C_3	SRR17129379	2,297,949	1011	9.9	785	98.8
Mock 22 °C_1	SRR17129385	2,408,614	1099	10.4	858	99.25
Mock 22 °C_2	SRR17129384	2,462,883	983	10.6	779	99.09
Mock 22 °C_3	SRR17129383	748,042	983	10.6	753	98.96

Reads N50-the shortest read at 50% of the total length of all reads; Median Q score-Median Pherd Quality Score; Median length (bp)-median read length, Aligned to the genome (%)-percent of reads mapped to the *Solanum tuberosum* genome reference sequence.

**Table 3:** Parameters of assembled transcriptomes.

	Number of Reads	Genes	Transcripts	Max Isoform Number per Gene	Isoforms per Gene	Mean Exon Number	Annotated Genes
Long reads	13,060,809	25,252	46,488	36	1.84	5.9	21,498
Short reads	1,267,036,962	25,646	47,174	53	1.84	6.4	21,398
Combined transcriptome	1,280,097,771	26,975	49,089	48	1.82	5.9	22,305

## Data Availability

The data supporting the findings of this study are available within the article and in the NCBI database, BioProject accession PRJNA786109.

## Supplementary Materials

The following supporting information can be downloaded at: https://www.mdpi.com/article/10.3390/plants11050635/s1, Table S1: Data on the assembled transcriptome, such as transcript location and functional annotation; Table S2: Data on the transcriptome, assembled from the long reads; Table S3: Log_2_FC values and functional annotation of all differentially expressed genes; Table S4: g:Profiler enrichment results, showing GO and KEGG terms enriched among differentially expressed genes; Table S5: Cluster IDs and annotation for differentially expressed genes; Table S6: g:Profiler enrichment results, showing GO and KEGG terms enriched in gene clusters from Figure 3; Table S7: Log_2_FC values of differentially expressed lncRNAs; Table S8: Log_2_FC expression values of differentially expressed antisense lncRNAs and corresponding mRNAs; Table S9: Table showing lncRNAs with miRNA target sites and the IDs of the miRNAs; Table S10: Isoforms with significant changes in ratio between Mock, 22 °C and PVY, 28 °C, and their functional annotation; Table S11: Results of RNA methylation analysis with m6Anet for virus Y sequence and xPore for mRNAs. Table S12: Primers used for quantitative RT PCR.
