# Supplementary material for: Transcriptomic Reprogramming, Alternative Splicing and RNA Methylation in Potato (Solanum tuberosum L.) Plants in Response to Potato Virus Y Infection
Source: Plants (Basel). 2022 Feb 25;11(5):635. doi: 10.3390/plants11050635 (PMC8912425; doi:10.3390/plants11050635)
Supplement: Supplementary file 1 [file plants-11-00635-s001.zip › Supplementary tables/Table.S12.pdf]

Table S12. Primers used for quantitative RT PCR

Primer	5′-3′ sequence	Reference	Primer Concentration (nM)	E (%)
PVY^{O}-F<br>PVY^{O}-R	TATGATGGATTTGGCGACCACTTGT<br>TAAACTAGGCAGCTCTGCATCATG	Makarova et al., 2018	400	95.7
StPARP1-F<br>StPARP1-R	GCCATGGAAAGCTGAGTATG<br>GCCACATCGGCATTAATCCATC	Soltu.DM03G032200*	400	95.4
StPARG-F<br>StPARG-R	AGGAAGAAATTCGATTTATGA<br>GAGGCATGCCTGGTATAACTCG	Soltu.DM.12G003820*	350	97.6
StADPR-PPase -F<br>StADPR-PPase -R	TATGATGACTTATTGGATTCC<br>CCATATGCCTGAAAGAGCCGACG	Soltu.DM.08G000850*	400	96.3
StSPDS-F<br>StSPDS-R	GATGGTTATTGATGTTTCAAGG<br>TGACATCATATGTTCCCGGAGC	Soltu.DM.06G014480*	400	95.9
StSAMDC-F<br>StSAMDC-R	GTTTCTGCCATCGGATTTGA<br>TTCATCCAATTGATCTTTGG	Soltu.DM02G030190*	400	96.3
StCOX-F<br>StCOX-R	GGTCGGACATACCTGAAAC<br>CCAAAAGTATGAAAAGCTGGAG	Baebler et al., 2011	350	97.3
StEF-1α-F<br>StEF-1α-R	CTTGACGCTCTTGACCAGATT<br>GAAGACGGAGGGGTTTGTCT	Nicot et al., 2005	350	98.7

Full references are provided in the main text. Primer concentrations giving the lowest threshold cycle (C_t) value were utilized in RT-PCR and are listed in the Table. E, efficiency of PCR amplification as calculated by CFX Manager Software. *Gene sequences were retrieved from Plant Genomics Resource Phytozome 12 (https://phytozome.jgi.doe.gov/pz/portal.html).
